# Comment on “New Method to Probe the Surface Properties of Polymer Thin Films by Two-Dimensional (2D) Inverse Gas Chromatography (iGC)”

**DOI:** 10.1021/acs.langmuir.4c02483

**Published:** 2024-10-25

**Authors:** Tayssir Hamieh

**Affiliations:** † Faculty of Science and Engineering, Maastricht University, P.O. Box 616, 6200 MD Maastricht, The Netherlands; ‡ Institut de Science des Matériaux de Mulhouse, Université de Haute-Alsace, CNRS, IS2M UMR 7361, F-68100 Mulhouse, France; § Laboratory of Materials, Catalysis, Environment and Analytical Methods Laboratory (MCEMA), Faculty of Sciences, Lebanese University, 961 Hadath, Lebanon

## Abstract

Determining London dispersive surface energy, polar thermodynamic variables, and Lewis acid–base behavior of solid surfaces is crucial in many industrial applications such as adhesion, catalysis, chemical engineering, biomaterials, and technologic processes. Inverse gas chromatography at infinite dilution is a powerful technique that allows the determination of the surface thermodynamic parameters of the interaction between solid materials and organic solvents. In their most recent study, Cho et al. determined the London dispersive surface energy and polar and Lewis acid–base parameters using the Schultz et al. method. These authors committed serious errors and inconsistencies. In this paper, we show the issues made by Cho et al. and proposed a more rigorous model to determine the surface properties of solid materials. Our model using the thermal effect on the surface area of organic molecules was applied on several solid surfaces and showed the various incoherences made by Cho et al. that also neglected the entropic contribution, while it was proved that this contribution is as important as the polar free energy of adsorption.

## Introduction

In their recent paper published in Langmuir, Cho et al.^1^ determined the London dispersive surface energy and the Lewis acid–base parameters of some polymers by varying some physicochemical properties and using the inverse gas chromatography (IGC) technique at infinite dilution. The authors used the method proposed by Schultz et al.^2^ based on the Fowkes relation^3^ expressed by eq 1
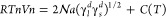
1where *T* is the temperature of solid material contained in the column, *R* is the perfect gas constant, *a* is the surface area of a molecule adsorbed on the solid materials, *V*_*n*_ is the net retention volume of the adsorbed solvent,  is Avogadro’s number, *C*(*T*) is a constant depending on temperature and solid surfaces, and γ_*l*_^*d*^and γ_*s*_^*d*^ are the respective dispersive components of the surface energy of the solvent and the solid. On the other hand, Cho et al.^1^ found a certain difficulty in determining the values of polar enthalpy (−Δ*H*_*a*_^*p*^) of adsorption of polar solvents because this required the measurements for every probe at least at three different temperatures. To determine the Lewis acid–base constants of polymers, the authors then proposed a particular method based the works of Voelkel et al.^4,5^ and Meyer et al.^6^ by assuming that (−Δ*H*_*a*_^*p*^) is proportional to the polar free Gibbs energy (−Δ*G*_*a*_^*p*^) of adsorption and neglecting the entropic contribution *T*(−Δ*S*_*a*_^*p*^).

However, Cho et al.^1^ committed several errors and inconsistencies in their approach. The first ones concerned the determination of the London dispersive surface energy of the materials. Indeed, these authors used the values of the surface area of *n*-alkanes of Kiselev utilized by Schultz et al.^2^ not only at ambient temperature but also at any other temperature. Hamieh et al.^7^ first proposed six molecular models for *n*-alkanes by using several ways to calculate the surface area of organic molecules which can be resumed as follows: van der Waals (VDW), Redlich-Kwong (R-K), Schultz/Kiselev, geometric, cylindrical, and spherical models. The values of the surface area of *n*-alkanes are given in Table 1 using the different molecular models.

**Table 1:** Surface Areas of Various Molecules (in Å^2^) Obtained Using the Various Models of van der Waals (VDW), Redlich-Kwong (R-K), Schultz/Kiselev, Geometric, Cylindrical, and Spherical^7^

Molecules	VDW	R-K	Schultz**/**Kiselev	Geometric	Cylindrical	Spherical
C_5_H_12_	47.0	36.8	45	32.9	39.3	36.4
C_6_H_14_	52.7	41.3	51.5	40.7	45.5	39.6
C_7_H_16_	59.2	46.4	57	48.5	51.8	42.7
C_8_H_18_	64.9	50.8	63	56.2	58.1	45.7
C_9_H_20_	69.6	54.5	69	64.0	64.4	48.7

The values in Table 1 clearly show that there is no universal model of surface area of *n*-alkanes that can be used to obtain an accurate value of the London dispersive surface energy γ_*s*_^*d*^ of solid surfaces. Several previous papers^8−14^ have shown a large difference in the values of γ_*s*_^*d*^ obtained by the different molecular models. The results showed a variation in the London dispersive surface energy of solids reaching more than 300% from one model to another.^13,14^

## Hamieh Thermal Model and Experimental Results

The disparities between the obtained results of γ_*s*_^*d*^ depending on the used molecular model led us to resolve this problem by proposing a new thermal model and studying the variations of the surface area *a*(*n*, *T*) of *n*-alkanes as a function of the temperature. A general expression^15^ of *a*(*n*, *T*) for *n*-alkanes was obtained
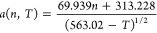
2where *n* is the carbon atom number of the *n*-alkane.

Another general expression for the surface area *a*_*X*_(*T*) of polar solvents was also proposed
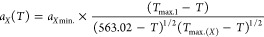
3where *a*_*X*_(*T*) and *a*_*Xmin*._ are respectively the corresponding surface areas expressed in Å^2^ and *T*_*max*.1_ and *T*_*max*. (*X*)_ are two new known intrinsic surface temperatures (in K) of polar molecules.^15^

Table 2 gives the different values of surface area of *n*-alkanes for different values of temperature obtained from the Hamieh thermal model.

**Table 2:** Values of the Surface Area *a*(*T*) (in Å^2^) of *n*-Alkanes versus Temperature *T* (in K)

*T*(K)	C5	C6	C7	C8	C9	C10
298.15	40.73	45.03	49.33	53.63	57.92	62.22
323.15	42.8	47.32	51.83	56.35	60.87	65.38
343.15	44.71	49.42	54.14	58.86	63.57	68.29
363.15	46.89	51.84	56.78	61.73	66.68	71.63
383.15	49.43	54.64	59.86	65.07	70.29	75.5
403.15	52.43	57.96	63.49	69.02	74.55	80.09
423.15	56.05	61.97	67.88	73.79	79.71	85.62
443.15	60.55	66.94	73.32	79.71	86.1	92.49
463.15	66.33	73.33	80.33	87.33	94.33	101.33

By comparing the results of the thermal model at 25 °C and the values used by Schultz et al.,^2^ one found a relative error varying from 10% for *n*-pentane to 20% for *n*-decane. These differences led to values of γ_*s*_^*d*^ that are very different.

To prove the difference between the various models and methods consisting of the determination of γ_*s*_^*d*^, one applied the above molecular models and Hamieh thermal model^15^ on different solid surfaces such as crystals of UiO-66, UiO-66-FA (using formic acid as a modulator), UiO-66(NH_2_), alumina, PMMA, and an oligoacenaphthylene/p-hydroxyphenylacetic acid (O-HPA) composite. The obtained results are listed in Table 3.

**Table 3:** Values of the London Dispersive Surface Energy γ_*s*_^*d*^(298.15 K) (mJ/m^2^) of Several Solid Surfaces Using Various Molecular Models

London dispersive surface energy γ_*s*_^*d*^(298.15 K) (mJ/m^2^)						
Models	UiO-66	UiO-66-FA	UiO-66(NH2)	Alumina	PMMA	O-HPA
Spherical	65.37	139.45	366.38	164.21	128.26	106.17
Hamieh-Gray	40.70	63.62	212.88	121.93	46.45	68.1
Redlich-Kwong	37.04	80.42	207.43	99.44	74.61	64.49
Hamieh model	38.56	82.02	214.39	90.79	50.68	41.62
Dorris-Gray	41.72	64.63	118.13	61.34	42.22	37.72
Cylindrical	23.52	50.67	130.16	60.31	43.48	33.66
VDW	22.65	48.22	127.04	60.07	45.73	39.28
Schultz/Kiselev	23.75	50.30	130.86	57.23	44.32	35.31
Geometric	17.85	39.02	98.63	43.46	32.58	24.89

Deviation percentage of the different methods and models						
Models	UiO-66	UiO-66-FA	UiO-66(NH2)	Alumina	PMMA	O-HPA
Spherical	69.5	70.0	70.9	80.9	153.1	155.1
Hamieh-Gray	5.5	–22.4	–0.7	34.3	–8.3	63.6
Redlich-Kwong	–3.9	–2.0	–3.2	9.5	47.2	54.9
Dorris-Gray	8.2	–21.2	–44.9	–32.4	–16.7	–9.4
Cylindrical	–39.0	–38.2	–39.3	–33.6	–14.2	–19.1
VDW	–41.3	–41.2	–40.7	–33.8	–9.8	–5.6
Schultz/Kiselev	–38.4	–38.7	–39.0	–37.0	–12.5	–15.2
Geometric	–53.7	–52.4	–54.0	–52.1	–35.7	–40.2

The results in Table 3 show that London dispersive surface energy γ_*s*_^*d*^ strongly depends on the chosen molecular model. It can be observed that the values of γ_*s*_^*d*^ obtained when using the spherical model are at least four times greater than those of the geometric model and about three times larger than the results obtained by the classic results of the Schultz/Kiselev method and used here by Cho et al.^1^ In fact, the thermal model gives the more accurate values of γ_*s*_^*d*^ of solid materials because it takes into consideration the effect of the temperature on the surface area of organic solvents and gives the variations of surface area *a*(*T*) as a function of temperature. The deviation between the results obtained by the various molecular models and IGC methods compared to those of the Hamieh thermal model reached in certain cases 150% and 40% when using the Schultz/Kiselev method.

It can be concluded from these results (Table 3) that the use of the Schultz/Kiselev method^2^ cannot be then applied to the determination of the polar free energy of adsorption of solvents and the Lewis acid–base constants of solid surfaces without taking into account the correction of the surface area of molecules due to the thermal effect.^15^

Furthermore, several chromatographic methods have been used since the 1970s to determine the polar interactions and the Lewis acid–base parameters of solid materials. These IGC methods used the representation of the free energy of adsorption Δ*G*_*a*_^0^(*T*) or *RT* ln *Vn*(*T*) of organic molecules adsorbed on solid particles as a function of thermodynamic. Several thermodynamic parameters were used in the literature:^16−22^ the boiling point *T*_*B*. *P*._ of the solvent probes proposed by Sawyer and Brookman,^16^ the vapor pressure *P*_0_ of the solvent proposed by Saint-Flour and Papirer,^17,18^ the deformation polarizability α_0_ used by Donnet et al.,^19^ the standard enthalpy of vaporization Δ*H*_*vap*._^0^ of solvents utilized by Chehimi et al.,^20^ and the topological index *χ*_*T*_ proposed by Brendlé and Papirer.^21,22^

In a recent paper,^15^ Hamieh proposed using the variations of the surface area *a*(*T*) against the temperature to correct not only the London dispersive surface energy of materials but also their polar interactions and Lewis acid–base parameters by applying the Fowkes relation. A new method more theoretically based on the London interaction equation was recently proposed by Hamieh^11,12^ to quantify the Lewis acid–base properties and polar free interaction energy. The determination of Δ*G*_*a*_^*p*^(*T*) of polar solvents leads to the specific enthalpy (−Δ*H*_*a*_^*p*^) and entropy (Δ*S*_*a*_^*p*^) of adsorbed molecules and therefore to the Lewis acid–base constants *K*_*A*_ and *K*_*D*_ of solids by using relation 4 first used by Papirer et al.^7,21,22^
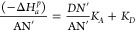
4where *AN*′ and *DN*′ respectively represent the electron donor and acceptor numbers of polar molecules determined by Gutmann^23^ and corrected by Riddle and Fowkes.^24^

Cho et al.^1^ used the classical method without considering the accurate values of the surface area of organic molecules. The values of the polar free energy and the Lewis acid–base constants or ratios obtained by Cho et al.^1^ cannot be considered as accurate. They have to be corrected using the new values of the surface areas of solvents.

As an example, we gave in Table 4 the results of the polar free energy Δ*G*_*a*_^*p*^(298.15 K), polar enthalpy (−Δ*H*_*a*_^*p*^), and entropic contribution (*T* × (−Δ*S*_*a*_^*p*^)) at 298.15 K for polar solvents (CH_2_Cl_2_, CHCl_3_, THF, diethyl ether, acetonitrile, toluene, and benzene) adsorbed on UiO-66 crystals with the percentages of the ratios [(298.15 × (−Δ*S*_*a*_^*p*^)) × 100/(−Δ*H*_*a*_^*p*^) and (−Δ*G*_*a*_^*p*^(298.15 K)) × 100/(−Δ*H*_*a*_^*p*^)] by applying the different molecular models and chromatographic methods.

**Table 4:** Values of the Polar Free Energy Δ*G*_*a*_^*p*^(298.15 K), Polar Enthalpy (−Δ*H*_*a*_^*p*^), and Entropic Contribution (*T* × (−Δ*S*_*a*_^*p*^)) at 298.15 K of the Various Polar Solvents Adsorbed on UiO-66 Crystals with the Percentages of the Ratios [(298.15 K × (−Δ*S*_*a*_^*p*^)) × 100/(−Δ*H*_*a*_^*p*^) and (−Δ_*a*_^*p*^(298.15 K)) × 100/(−Δ*H*_*a*_^*p*^)] for the Different Molecular Models and Chromatographic Methods

(−Δ*G*_*a*_^*p*^(298.15 K)) (kJ/mol)							
Molecular or IGC model	CH_2_Cl_2_	CHCl_3_	THF	Diethyl ether	Acetonitrile	Toluene	Benzene
Schultz/Kiselev	4.056	3.761	4.031	18.581	10.821	–0.699	–0.585
Spherical	–8.343	–9.040	7.487	17.312	15.524	–0.174	–0.193
Geometric	10.654	9.642	15.546	10.278	4.749	1.274	–0.233
van der Waals	–1.679	–9.051	32.600	–1.938	7.235	7.838	1.183
Redlich–Kwong	6.547	2.835	5.289	20.713	0.159	4.168	–1.476
Cylindrical	3.194	3.053	2.878	15.781	12.908	–1.175	–1.425
Hamieh model	1.430	2.622	26.338	5.254	8.609	3.256	1.550
Topological index	9.553	7.092	23.719	5.307	28.445	4.195	0.664
Deformation polarizability	4.186	2.530	29.257	5.528	29.926	4.136	1.920
Vapor pressure	3.607	2.504	24.411	25.063	24.027	5.570	0.994

(−Δ*H*_*a*_^*p*^) (kJ/mol)							
Molecular or IGC model	CH_2_Cl_2_	CHCl_3_	THF	Diethyl ether	Acetonitrile	Toluene	Benzene
Schultz/Kiselev	12.345	11.662	52.331	47.800	39.145	8.544	1.800
Spherical	26.153	13.083	53.700	53.090	45.339	4.000	1.000
Geometric	30.213	27.233	43.870	27.570	23.778	6.241	0.303
van der Waals	24.349	16.172	92.230	33.840	21.606	27.650	3.300
Redlich–Kwong	24.436	16.252	67.900	55.000	21.626	15.200	2.400
Cylindrical	10.230	9.821	44.231	43.211	39.145	6.875	0.751
Hamieh model	14.250	13.654	44.674	25.230	16.450	5.803	4.740
Topological index	10.000	8.076	35.645	7.394	40.520	8.668	1.260
Deformation polarizability	5.230	3.365	38.500	10.000	42.597	8.608	1.920
Vapor pressure	4.800	3.100	30.970	31.643	29.480	7.020	1.056

(298.15 × (−Δ*S*_*a*_^*p*^)) (kJ/mol)							
Molecular or IGC model	CH_2_Cl_2_	CHCl_3_	THF	Diethyl ether	Acetonitrile	Toluene	Benzene
Schultz/Kiselev	8.289	7.901	48.300	29.219	28.324	9.243	2.385
Spherical	34.496	22.123	46.213	35.778	29.815	4.174	1.193
Geometric	19.559	17.591	28.324	17.293	19.029	4.967	0.537
van der Waals	26.028	25.223	59.630	35.778	14.371	19.812	2.117
Redlich-Kwong	17.889	13.417	62.612	34.287	21.467	11.032	3.876
Cylindrical	7.036	6.768	41.353	27.430	26.237	8.050	2.176
Hamieh model	12.820	11.032	18.336	19.976	7.841	2.546	3.190
Topological index	0.447	0.984	11.926	2.087	12.075	4.472	0.596
Deformation polarizability	1.044	0.835	9.243	4.472	12.671	4.472	0.000
Vapor pressure	1.193	0.596	6.559	6.579	5.453	1.449	0.062

(298.15 × (−Δ*S*_*a*_^*p*^)) *×* 100/(−Δ*H*_*a*_^*p*^)							
Molecular or IGC model	CH_2_Cl_2_	CHCl_3_	THF	Diethyl ether	Acetonitrile	Toluene	Benzene
Schultz/Kiselev	67.14	67.75	92.30	61.13	72.36	108.18	132.51
Spherical	131.90	169.10	86.06	67.39	65.76	104.35	119.26
Geometric	64.74	64.59	64.56	62.72	80.03	79.58	176.94
van der Waals	106.90	155.97	64.65	105.73	66.51	71.65	64.15
Redlich–Kwong	73.21	82.55	92.21	62.34	99.26	72.58	161.50
Cylindrical	68.78	68.91	93.49	63.48	67.03	117.09	289.81
Hamieh model	89.97	80.79	41.04	79.18	47.67	43.88	67.30
Topological index	4.47	12.18	33.46	28.23	29.80	51.60	47.33
Deformation polarizability	19.95	24.81	24.01	44.72	29.75	51.95	0.00
Vapor pressure	24.85	19.24	21.18	20.79	18.50	20.64	5.91

(−Δ*G*_*a*_^*p*^(298.15 K)) *×* 100/(−Δ*H*_*a*_^*p*^)							
Molecular or IGC model	CH_2_Cl_2_	CHCl_3_	THF	Diethyl ether	Acetonitrile	Toluene	Benzene
Schultz/Kiselev	32.86	32.25	7.70	38.87	27.64	–8.18	–32.51
Spherical	–31.90	–69.10	13.94	32.61	34.24	–4.35	–19.26
Geometric	35.26	35.41	35.44	37.28	19.97	20.42	–76.94
van der Waals	–6.90	–55.97	35.35	–5.73	33.49	28.35	35.85
Redlich-Kwong	26.79	17.45	7.79	37.66	0.74	27.42	–61.50
Cylindrical	31.22	31.09	6.51	36.52	32.97	–17.09	–189.81
Hamieh model	10.03	19.21	58.96	20.82	52.33	56.12	32.70
Topological index	95.53	87.82	66.54	71.77	70.20	48.40	52.67
Deformation polarizability	80.05	75.19	75.99	55.28	70.25	48.05	100.00
Vapor pressure	75.15	80.76	78.82	79.21	81.50	79.36	94.09

The results presented in Table 4 clearly show a large difference in the values of the polar free energy, enthalpy, and entropy of the different polar solvents adsorbed on UiO-66 crystals strongly depending on the used IGC methods or models. Very large disparities in these values were observed. Only the results obtained by the thermal model can be more accurate.

On the other hand, Cho et al.^1^ assumed in their paper that (−Δ*G*_*a*_^*p*^) and (−Δ*H*_*a*_^*p*^) are proportional and considered that (−Δ*G*_*a*_^*p*^) ≅ (−Δ*H*_*a*_^*p*^) and used the following relation
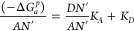
5to deduce the acid–base constants *K*_*A*_ and *K*_*D*_ or their ratio.

However, the approximation utilized by Cho et al.^1^ is definitely wrong. Indeed, for all organic solvents adsorbed on different solid substrates, the entropic contribution was proven to be as important as the enthalpic contribution. The results in Table 4 proved that the entropic contribution cannot be neglected as it was assumed by Cho et al.^1^ The determination of the percentages [(298.15 × (−Δ*S*_*a*_^*p*^)) × 100/(−Δ*H*_*a*_^*p*^) and (−Δ*G*_*a*_^*p*^(298.15 K)) × 100/(−Δ*H*_*a*_^*p*^)] in Table 4 showed values higher than 100% for many models and chromatographic methods and for the various solid materials. It can be easily observed in Table 4 that the values of the entropic contribution for all polar solvents are as important as the polar free energy, which is also shown to be lower than the polar enthalpy of adsorbed solvent.

The difference in the values of the polar free energy and enthalpy of adsorption of polar solvents on UiO-66 crystals given in Table 4 leads to different values of the acid–base constants, depending on the chosen models and IGC methods. The calculated values of the Lewis acid *K*_*A*_ and base *K*_*D*_ constants are presented in Table 5.

**Table 5:** Values of the Lewis Acid–Base Constants of UiO-66 Crystals and Their Acid–Base Ratios for the Different Molecular Models and IGC Methods

Values of the Lewis acid–base parameters			
Models and IGC methods	*K*_*A*_	*K*_*D*_	*K*_*A*_/*K*_*D*_
Schultz/Kiselev	0.600	0.404	1.485
Spherical	0.581	0.473	1.230
Geometric	0.493	0.146	3.368
van der Waals	1.014	0.190	5.345
Redlich-Kwong	0.772	0.469	1.646
Cylindrical	0.517	0.249	2.078
Hamieh model	0.490	0.292	1.678
Topological index	0.380	0.170	2.233
Deformation polarizability	0.412	0.204	2.021
Vapor pressure	0.357	0.336	1.063
Average values	0.56	0.29	1.915

Deviation percentage of the Lewis acid–base parameter			
Models and IGC methods	*K*_*A*_	*K*_*D*_	*K*_*A*_/*K*_*D*_
Schultz/Kiselev	18.6	62.0	–26.7
Spherical	0.6	–50.0	100.7
Geometric	106.9	–34.9	218.5
van der Waals	57.6	60.6	–1.9
Redlich-Kwong	5.5	–14.7	23.8
Cylindrical	–22.4	–41.8	33.1
Topological index	–15.9	–30.1	20.4
Deformation polarizability	–27.1	15.1	–36.7
Vapor pressure	14.3	–0.7	14.1

Table 5 also shows different values of the Lewis acid and base constants of the UiO-66 solid material. The unique and interesting qualitative result shown in Table 5 is the higher acidity of the above solid substrate relative to its basicity. The most accurate result is that obtained by the Hamieh thermal model taking into account the effect of the temperature on the surface area of organic molecules. Table 5 also gave the deviation percentage between the acid–base constants obtained by the different chromatographic methods compared to the results obtained by the thermal model. It can be observed (Table 5) that this deviation reached 60% when using the Schultz/Kiselev method, thus proving the nonvalidity of this method. In the next section, the surface properties of PMMA were also determined to show the highest difference between the different IGC methods.

## Surface Properties of PMMA

**Figure 1:**
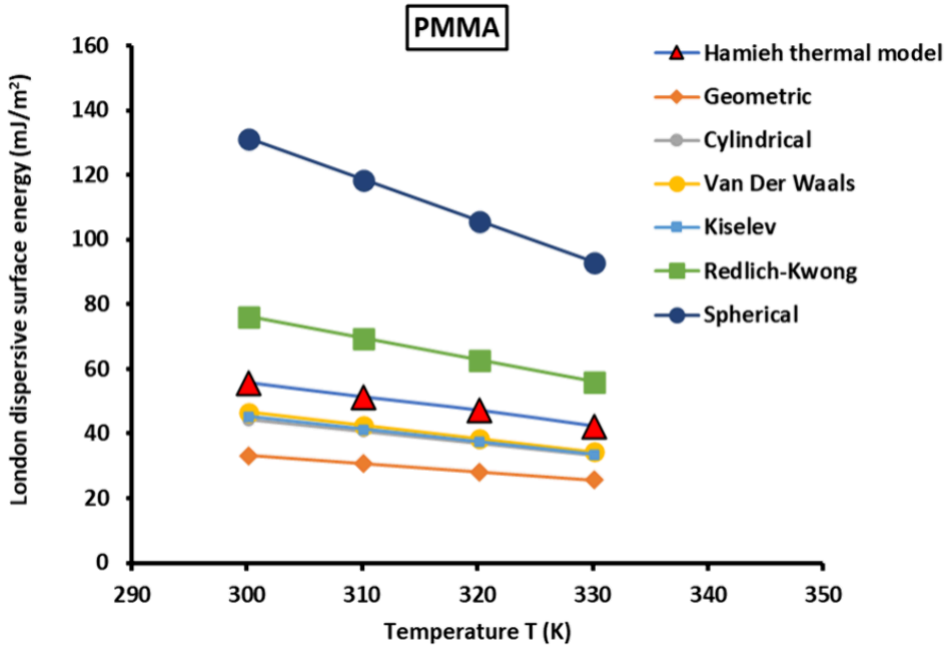
Variations of γ_*s*_^*d*^(*T*) of PMMA particles as a function of the temperature using the various molecular models compared with the Hamieh thermal model.

To compare the results obtained by the classical methods and molecular models to those of our new methodology taking into account the thermal effect, we studied the surface thermodynamic properties of PMMA by inverse gas chromatography at infinite dilution at temperatures lower than those of beta-relaxation and the glass transition of PMMA. The values of the London dispersion surface energy of PMMA at different temperatures are plotted in Figure 1.

It is shown in Figure 1 that the values of γ_*s*_^*d*^(*T*) of PMMA particles strongly depend on the method or model chosen. The geometric molecular model gave the lowest dispersive surface energy, while the spherical model gave the highest values. A deviation of 20% was observed between the results of the Schultz method (using the Kiselev model) and those of the thermal Hamieh model certainly due to the temperature effect on the surface area of *n*-alkanes. This led to lower values of γ_*s*_^*d*^(*T*) of PMMA obtained by Cho et al.^1^ because of the above thermal effect neglected by the Schultz method. The deviation observed between the variations of the γ_*s*_^*d*^(*T*) of PMMA as a function of temperature is shown in Table 6.

**Table 6:** Expressions of γ_*s*_^*d*^(*T*) of PMMA by Applying the Different Molecular Models and IGC Methods

IGC method or model	London dispersive surface energy
Hamieh thermal model	γ_*s*_^*d*^(*T*) = −0.443*T* + 188.6
Geometric	γ_*s*_^*d*^(*T*) = −0.247*T* + 107.1
Cylindrical	γ_*s*_^*d*^(*T*) = −0.363*T* + 153.1
van der Waals	γ_*s*_^*d*^(*T*) = −0.41*T* + 169.73
Schultz/Kiselev	γ_*s*_^*d*^(*T*) = −0.389*T* + 162.0
Redlich-Kwong	γ_*s*_^*d*^(*T*) = −0.669*T* + 276.9
Spherical	γ_*s*_^*d*^(*T*) = −1.277*T* + 514.4

**Figure 2:**
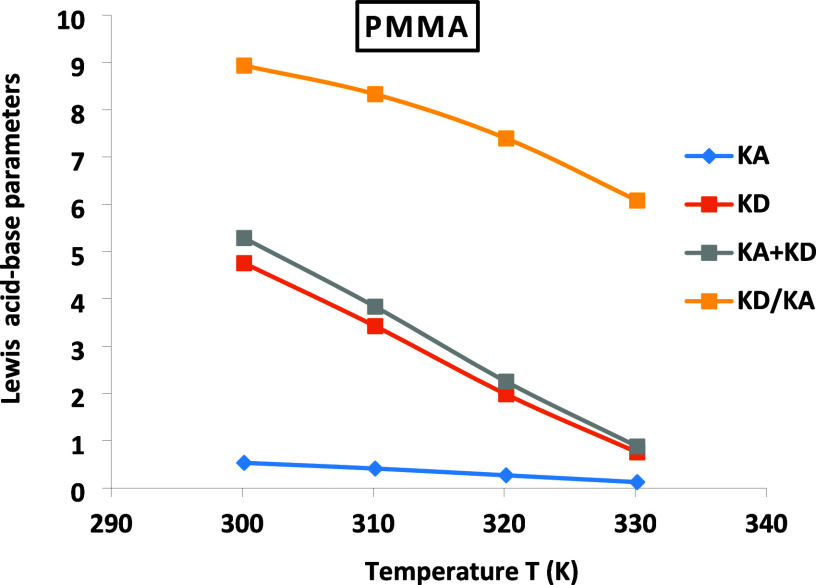
Variations of the Lewis acid–base parameters *K*_*A*_, *K*_*D*_, *K*_*D*_/*K*_*A*_, and *K*_*D*_ + *K*_*A*_ of PMMA particles.

Table 6 shows large variations of the slope and the ordinate at the origin depending on the method used. On the other hand, the application of our model gave an important variation of the Lewis acid–base parameters of PMMA as a function of temperature (Figure 2), which also showed the highest basicity.

The results in Figure 2 clearly show the highest basic character of PMMA (about 9 times more basic than acidic at 300.15 K). A linear decrease in the Lewis acid–base character was observed when the temperature increased. The linear straight lines of *K*_*A*_, *K*_*D*_, and *K*_*D*_/*K*_*A*_ shown in Figure 2 led to the different expressions given in Table 7 as a function of the temperature.

**Table 7:** Expressions of *K*_*A*_(*T*), *K*_*D*_(*T*), *K*_*D*_ + *K*_*A*_(*T*), and *K*_*D*_/*K*_*A*_(*T*) of PMMA by Using the Thermal Model

Lewis acid–base parameters	Expression *X*(*T*)	*R*^2^
Lewis acid parameter	*K*_*A*_(*T*) = −0.014*T* + 4.644	0.9984
Lewis base parameter	*K*_*D*_(*T*) = −0.135*T* + 45.11	0.9991
Lewis acid–base parameter	*K*_*D*_ + *K*_*A*_(*T*) = −0.149*T* + 49.754	0.9993
Lewis acid–base ratio	*K*_*D*_/*K*_*A*_(*T*) = −0.095*T* + 37.634	0.9729

The different expressions given in Table 7 clearly show the important effect of the temperature on the Lewis acid–base character of PMMA. This dependence of these acid–base parameters on the temperature was not highlighted when using the classical Schultz method.

These results then require a correction of the surface thermodynamic parameters obtained by Cho et al., particularly that of the London dispersive surface energy of polymers and their Lewis acid–base constants.

## Validation of the Thermal Model Results by Using the London Dispersion Equation

In a recent work,^11^ we proposed a new method to determine the free dispersive and polar energies of materials and the Lewis acid–base parameters as a function of temperature. This method was based on the London dispersive interaction energy Δ*G*_*a*_^*d*^ given by eq 6
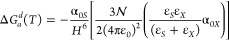
6where ε_0_ is the dielectric constant of vacuum, α_0*S*_ and α_0*X*_ are the respective deformation polarizabilities of the solid material denoted by S and the organic solvent denoted by X, separated by a distance *H*, and *ε*_*S*_ and *ε*_*X*_ are their corresponding ionization energies.

In the case of polar organic molecules adsorbed on the solid materials, one can write the free energy Δ*G*_*a*_^0^ of adsorption
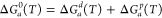
7where Δ*G*_*a*_^*p*^(*T*) represents the free polar energy of the polar solvents.

By choosing a new chromatographic interaction parameter  given by
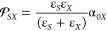
8the free polar energy Δ*G*_*a*_^*p*^(*polar*) of polar molecules adsorbed on solid surfaces can be written as follows
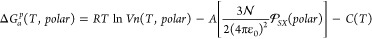
9where *A* is the slope of the nonpolar straight line given by
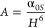
10

Using this new methodology, we are able to determine the polar and dispersive free energies of adsorption as well as the Lewis acid–base parameters of PMMA as a function of the temperature. The various solvents used in this study were the following: *n*-alkanes (from *n*-pentane to *n*-nonane) and polar solvents such as dichloromethane, chloroform, and carbon tetrachloride (Lewis acid solvents), ethyl acetate, diethyl ether, and tetrahydrofuran (Lewis base solvents), and toluene (Lewis amphoteric solvent). The results of the surface parameters of adsorbed organic molecules are given in Table 8.

**Table 8:** Values of the Dispersive Δ*G*_*a*_^*d*^(*T*), Polar Δ*G*_*a*_^*p*^(*T*), and Total Free Δ*G*_*a*_^0^(*T*) Energies (in kJ/mol) of the Organic Solvents Adsorbed on PMMA Particles at Different Temperatures

Dispersive free energy of adsorption Δ*G*_*a*_^*d*^(*T*) (kJ/mol)				
*T*(K)	300.15	310.15	320.15	330.15
*n*-Pentane	17.233	15.861	15.193	15.231
*n*-Hexane	17.862	17.508	15.872	12.955
*n*-Heptane	20.262	19.861	18.006	14.698
*n*-Octane	23.529	23.062	20.908	17.065
*n*-Nonane	25.609	25.103	22.761	18.583
CCl_4_	17.161	16.822	15.253	12.455
CH_2_Cl_2_	11.340	11.115	10.078	8.228
Chloroform	13.950	13.674	12.395	10.116
Diethyl ether	13.845	13.572	12.306	10.048
THF	11.931	11.694	10.602	8.653
Ethyl acetate	13.687	13.416	12.164	9.931
Toluene	16.680	16.350	14.825	12.103

Polar free energy of adsorption Δ*G*_*a*_^*p*^(*T*) (kJ/mol)				
*T*(K)	300.15	310.15	320.15	330.15
*n*-Pentane	0	0	0	0
*n*-Hexane	0	0	0	0
*n*-Heptane	0	0	0	0
*n*-Octane	0	0	0	0
*n*-Nonane	0	0	0	0
CCl_4_	15.160	14.764	14.929	15.653
CH_2_Cl_2_	21.601	19.986	19.051	18.796
Chloroform	16.116	14.531	14.266	15.321
Diethyl ether	13.403	11.696	11.549	12.963
THF	22.969	22.001	21.833	22.465
Ethyl acetate	17.413	15.932	15.131	15.010
Toluene	11.981	10.815	9.869	9.144

Total free energy of adsorption Δ*G*_*a*_^0^(*T*) (kJ/mol)				
*T*(K)	300.15	310.15	320.15	330.15
*n*-Pentane	17.233	15.861	15.193	15.231
*n*-Hexane	17.862	17.508	15.872	12.955
*n*-Heptane	20.262	19.861	18.006	14.698
*n*-Octane	23.529	23.062	20.908	17.065
*n*-Nonane	25.609	25.103	22.761	18.583
CCl_4_	32.321	31.586	30.182	28.108
CH_2_Cl_2_	32.940	31.101	29.129	27.025
Chloroform	30.067	28.204	26.661	25.437
Diethyl ether	27.248	25.268	23.855	23.010
THF	34.900	33.696	32.435	31.119
Ethyl acetate	31.100	29.348	27.295	24.941
Toluene	28.661	27.165	24.694	21.247

The results in Table 8 show the highest basic character of PMMA due to the strongest polar interaction between the Lewis acid solvents and the PMMA particles. The values of the enthalpic and entropic contributions of the adsorbed polar solvents were determined at 300.15 K and are given in Table 9 with the percentages of the ratios of the entropic contribution *T*Δ*S*_*a*_^*p*^ to the enthalpy Δ*H*_*a*_^*p*^ and to the total free polar energy Δ*G*_*a*_^*p*^ of adsorption.

**Table 9:** Values of the Entropic *T*Δ*S*_*a*_^*p*^ and Enthalpic Δ*H*_*a*_^*p*^ Contributions, the Total Free Polar Energy Δ*G*_*a*_^*p*^ of Adsorption, and the Corresponding Percentages *T*Δ*S*_*a*_^*p*^/Δ*H*_*a*_^*p*^ (in %) and *T*Δ*S*_*a*_^*p*^/Δ*G*_*a*_^*p*^ (in %) at 300.15 K

Solvents	*T*Δ*S*_*a*_^*p*^	Δ*H*_*a*_^*p*^	Δ*G*_*a*_^*p*^	*T*Δ*S*_*a*_^*p*^/Δ*H*_*a*_^*p*^ (in %)	*T*Δ*S*_*a*_^*p*^/Δ*G*_*a*_^*p*^ (in %)
CCl4	27.034	11.886	15.160	44.0	78.4
CH_2_Cl_2_	70.069	48.474	21.601	69.2	224.4
Chloroform	63.696	47.574	16.116	74.7	295.2
Diethyl ether	64.626	51.236	13.403	79.3	382.3
THF	52.024	29.055	22.969	55.8	126.5
Ethyl acetate	61.859	44.452	17.413	71.9	255.3
Toluene	46.969	34.997	11.981	74.5	292.1

It can be observed in Table 9 that the ratios *T*Δ*S*_*a*_^*p*^/Δ*H*_*a*_^*p*^ and *T*Δ*S*_*a*_^*p*^/Δ*G*_*a*_^*p*^ in the case of PMMA are not negligible and they are higher than 44%, proving that the approximation used by Cho et al.^1^ by neglecting the entropic contribution cannot be justified.

Table 10 gives the Lewis acid–base constants of PMMA as a function of the temperature by using the new methodology.

**Table 10:** Values of *K*_*A*_(*T*), *K*_*D*_(*T*), *K*_*D*_ + *K*_*A*_(*T*), and *K*_*D*_/*K*_*A*_(*T*) of PMMA at Different Temperatures and the Corresponding Expressions, Using the London Dispersion Interaction Energy

*T*(K)	300.15	310.15	320.15	330.15	Equation	*R*^2^
*K*_*A*_	0.54	0.421	0.271	0.131	*K*_*A*_(*T*) = −0.014*T* + 4.680	0.998
*K*_*D*_	4.802	3.464	1.972	0.784	*K*_*D*_(*T*) = −0.136*T* + 45.446	0.9982
*K*_*A*_ + *K*_*D*_	5.342	3.885	2.243	0.915	*K*_*A*_ (*T*) + *K*_*D*_ (*T*) = −0.149*T* + 50.126	0.9985
*K*_*D*_/*K*_*A*_	8.893	8.228	7.277	5.985	*K*_*D*_/*K*_*A*_(*T*) = −0.0967*T* + 38.086	0.9794

The comparison between Tables 8 and 10 led to identical results, thus proving that the values obtained by the new applied method using the London dispersion equation (Table 10) confirmed those obtained by applying the thermal model (Table 8). This validation of the Hamieh thermal model by another rigorous scientific method once again showed the nonvalidity of the Schultz method that did not take into account the temperature effect on the surface area of organic molecules.

## Conclusions

In this study, it was proven that the Schultz et al. method cannot be used for an accurate determination of the London dispersive surface energy, the polar free energy, and the Lewis acid–base parameters. The nonvalidity of this method was demonstrated, and the Hamieh thermal model consisted of the correction of the values of the surface area of organic molecules by giving their expressions as a function of temperature. Accurate values of the London dispersive surface energy and Lewis acid–base constants were obtained. The new thermal model was applied on different solid surfaces such as crystals of UiO-66, UiO-66-FA, UiO-66(NH_2_), alumina, PMMA, and the oligoacenaphthylene/p-hydroxyphenylacetic acid (O-HPA) composite. It was proven that the deviation between the results obtained by the Schultz method compared to those of the Hamieh thermal model reached in certain cases 40%. The determination of the polar free enthalpy and entropy of the various polar solvents adsorbed on the different solid surfaces as a function of the temperature clearly showed that the values of the entropic contribution (*T* × (−Δ*S*_*a*_^*p*^)) were proven to be twice as high as those of the enthalpy of adsorption (−Δ*H*_*a*_^*p*^) in many cases and especially in the case of PMMA at different temperatures. However, Cho et al. neglected the entropic contribution, and therefore the Lewis acid–base constants obtained by this method cannot be considered to be accurate. Our results proved that the Lewis acid–base parameters strongly depend on the temperature, and an important deviation of the results of Cho et al. was proven. A new method based on the London dispersion interaction energy was used. The results obtained by this new methodology confirmed the validity of the Hamieh thermal model by showing identical quantitative results of the Lewis acid–base parameters of PMMA as a function of the temperature.

This new study then constitutes a new contribution, leading to the correction of the surface thermodynamic properties of surface solids, and can be used in several industrial applications and adhesion processes.
